# An apparent primitive mass of the mesentery: A case report

**DOI:** 10.1097/MD.0000000000029464

**Published:** 2022-06-17

**Authors:** Antonio Costanzo, Marco Canziani, Cesare Carlo Ferrari, Valentina Bertocchi, Saro Cutaia, Eraldo Oreste Bucci, Elisabetta Uslenghi, Andrea Ferretti, Marco De Luca, Fabio Ceriani

**Affiliations:** a General Surgery Department, IRCCS MultiMedica, Italy; b Medical Oncology Department, IRCCS MultiMedica, Italy; c Radiology Department, IRCCS MultiMedica, Italy; d Inter-Hospital Division of Pathology, IRCCS MultiMedica, Castellanza (Va) Italy.

**Keywords:** case report, computed tomography, diabetes mellitus, 68Ga-positron emission tomography, neuroendocrine tumour, primary mesenteric NET

## Abstract

**Introduction:**

Neuroendocrine tumours (NETs) are rare tumors. 55% of NETs originate in the gastrointestinal tract and the liver is the most common site of distant metastases. Serum chromogranin A is the most common biomarker for assessing the extent of disease and monitoring treatment; carcinoid syndrome occurs in 19% of NETs and is characterized by chronic diarrhea or flushing. Primary mesenteric NETs are rare and have been described only in case reports in literature; our case is an apparent primary mesenteric NETs with a surgical program to remove the mesenteric mass and subrenal interaortocaval and retrocaval lymphadenectomies.

**Patient concerns:**

A 73-year old man came to us because he had been experiencing abdominal pain for a year and he had recently developed diabetes mellitus. He was an active smoker with arterial hypertension.

**Diagnosis:**

After a computed tomography scan and 68 Gallium-positron emission tomography, a diagnosis of what appeared to be a primary mesenteric NET with retrocaval and interaortocaval lymph nodes was made. Laparoscopic biopsy showed NET G2 positive for serotonin, chromogranin A, synaptophysin.

**Interventions:**

The intraoperative finding of a primitive ileum-NET changed the surgical program. We removed the mesenteric mass with the lymph nodes of the superior mesenteric vessel and the middle distal ileum along with the cecum.

**Outcomes:**

The postoperative course was normal, and the patient was discharged on the seventh postoperative day without signs of short bowel syndrome. Follow-up at 6 months revealed no evidence of short bowel syndrome or disease progression.

**Conclusion:**

68 Gallium-positron emission tomography does not show NETs smaller than 0.5 mm. Accurate palpation of the intestine is essential during surgery for NETs for two reasons: to find the primitive, and because of the risk of multiple intestinal primitives.

## 1 Introduction

NETs (neuroendocrine tumors) are rare tumors, with an estimated incidence of 3.56/100 000/year in the United States and 1.33 to 2.33/100 000/year in Europe. NETs can be sporadic or, in rare cases, are associated with multiple endocrine neoplasia 1, neurofibromatosis type 1, and von Hippel-Lindau syndrome. The average age of the patients with NETs is 64 years.^[1]^

A high percentage (55%) of NETs arise in the gastrointestinal tract, accounting for 2% of all gastrointestinal tumors; 45% of gastrointestinal tumors originate in the small intestine.

The liver is the most common site of distant metastases, while the peritoneum and lungs are the least common. Other possible sites of origin include the lungs, thymus, ovaries and kidneys. NETs arise from enterochromaffin-like cells that contain chromogranin A, synaptophysin, and neuron-specific enolase. Serum chromogranin A is the most common biomarker for assessing the extent of disease and monitoring treatment and is elevated in both functioning and non-functioning NETs; most gastrointestinal NETs are non-functioning. In 2019, the World Health Organization updated the classification of NETs: NET G1-G2-G3 and NEC G3 (neuroendocrine carcinoma Grade 3). The difference between these three types of NETs is based on the mitotic index and Ki-67, and the difference between NET G3 and NEC G3 is related to the mutation status of the tumor protein 53 and protein retinoblastoma proteins.^[1]^ Carcinoid syndrome occurs in 19% of NETs and is characterized by chronic diarrhea or flushing resulting from the secretion of serotonin, other bioactive amines, and peptides by the tumor intosystemic circulation. This pattern is typical of liver metastasis. The diagnosis of carcinoid syndrome is based on elevated serum chromogranin A level and 24-hour urinary excretion of 5-hydroxyindoleacetic acid. The mesentery may be affected by direct infiltration from the primary mass in the intestine or by lymphohematogenous spread; however primary mesenteric NETs (PMNETs) have rarely been reported.^[2–5]^ This case report follows the case report guidelines.^[6]^

## 2 Case presentation

A 73-year-old man presented to us because he had been suffering from abdominal pain for a year and had recently developed diabetes mellitus. His family history was negative for neoplasia and he was an active smoker with arterial hypertension. Gastroscopy and colonoscopy were normal, as was abdominal computed tomography (CT) with contrast performed 6 months earlier.

The patient presented only a slight tenderness on deep palpation in mesogastrium, without other clinical findings.

**Figure 1:**
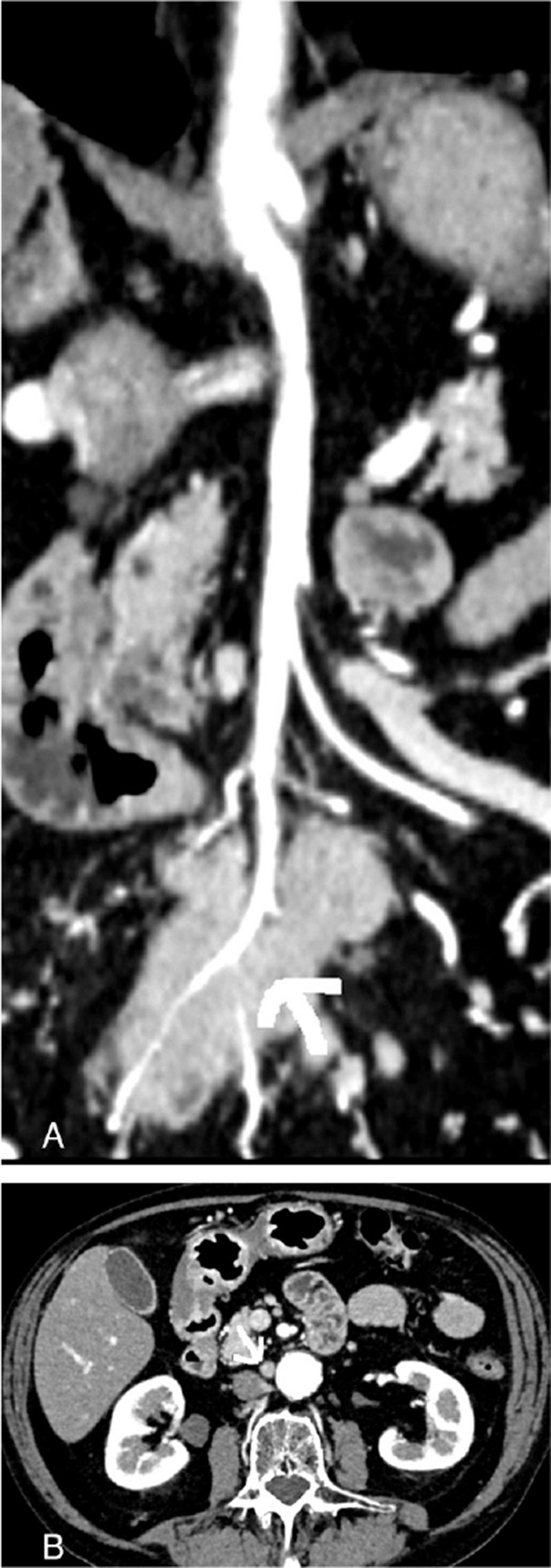
(A) Coronal view CT of tumour (arrow) with vascular reconstruction: encased distal branches of superior mesenteric artery. (B) CT view of interaortocaval lymph node (arrow): enlarged node with short axis of 11 mm. CT = computed tomography.

We arranged for a chest-abdominal CT with contrast, which revealed a 47 × 30 × 52 mm mesenteric mass consistent with lymphadenopathy and a subrenal interaortocaval lymph node of 11 mm. Computed tomography revealed no other localization of the disease. The mesenteric mass encased the distal portion of the superior mesenteric artery; CT findings are shown in Figure 1. Serum lactate dehydrogenase, carcinoembryonic antigen. Ca 19-9, blood count, and renal and liver functions were normal. The patient underwent exploratory laparoscopy with contextual biopsy and a normal postoperative course. Histologicale examination revealed a NET G2 positive for serotonin, chromogranin A, synaptophysin, and insulinoma associated protein 1, and focally for p53; it was negative for Nkx3.1, thyroid transcription factor 1, paired box 8, gastrin, glucagon, insulin, somatostatin, and pancreatic polypeptide, while the value for Ki-67 was 6.5%. Immunoreactivity for retinoblastoma was not evaluable.

**Figure 2:**
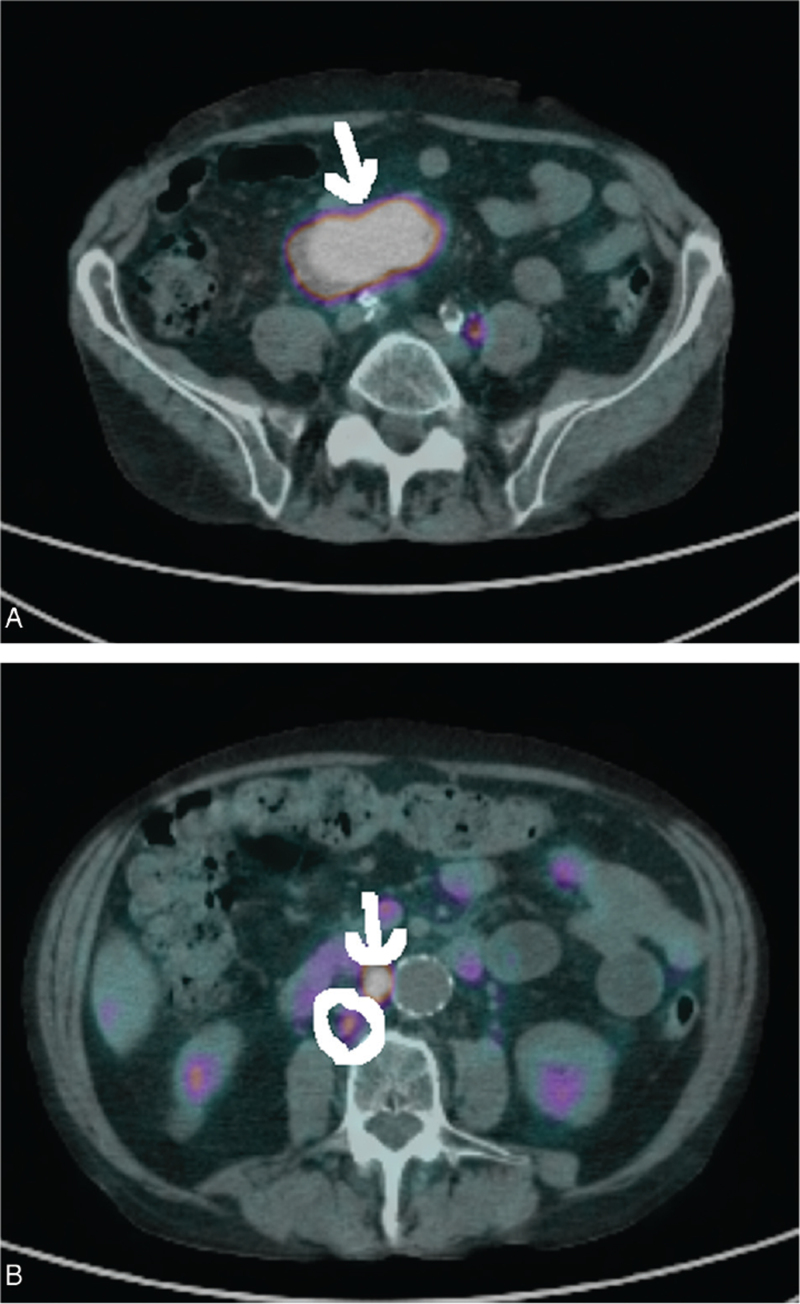
(A) Ga PET DOTATOC mesenteric tumour (arrow). (B) Ga PET DOTATOC subrenal retrocaval (circle) and interaortocaval lymph node (arrow): lymph nodes with pathologic SUV. Ga-PET = Gallium-positron emission tomography, SUV = standardized uptake value.

We performed 68Ga DOTATOC PET (185 megabecquerel) with contrast fixation of the mesenteric mass and two lymph nodes, one retrocaval and one interaortocaval. The PET findings are shown in Figure 2.

We used this type of PET because it is more sensitive than the In-DTPA-octreotide scan as described by Deppen et al.^[7]^ The patient showed no signs of carcinoid syndrome: serum chromogranin A level was 242.6 μg/L (normal value: 0–100) and urinary hydroxyindoleacetic acid level was 3.2 mg/24 h (normal value: 0–6.9).

**Figure 3:**
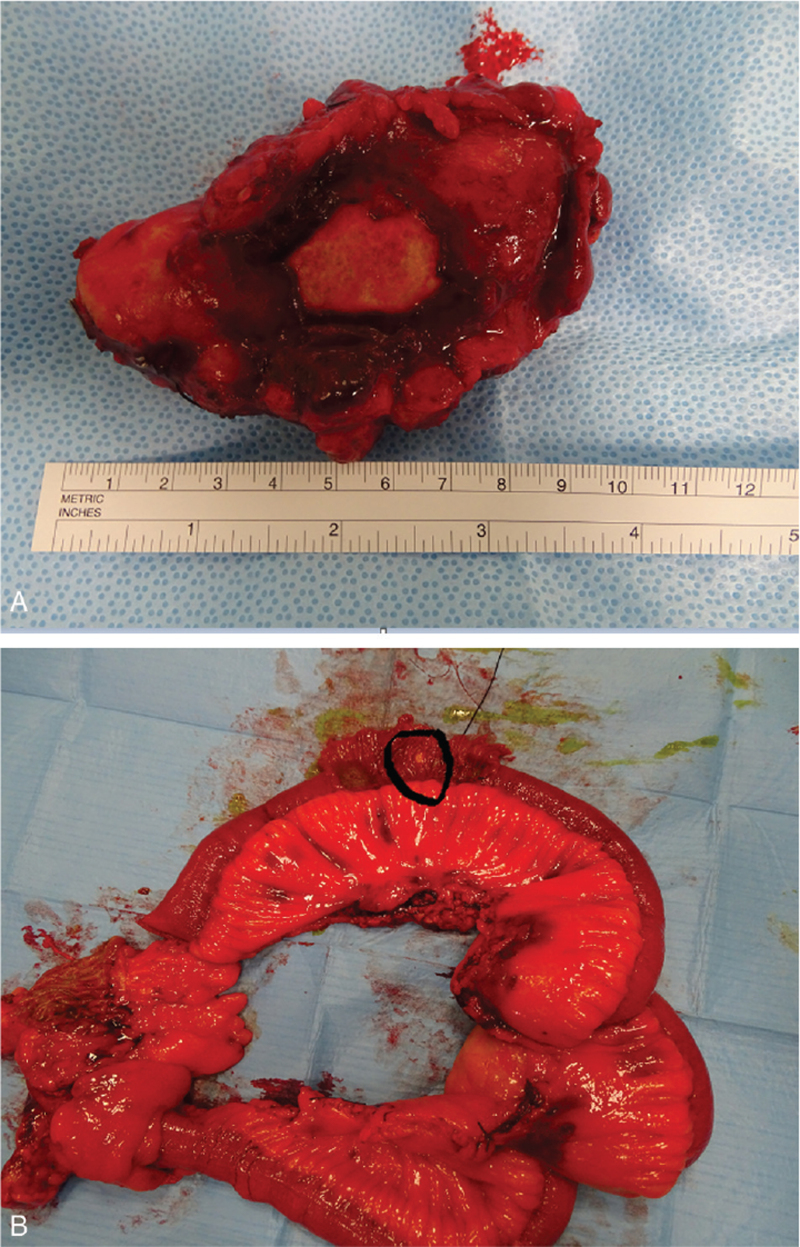
(A) Mesenteric mass en bloc with lymph nodes: 10 × 5 cm mass with fused mesenteric lymph nodes. (B) Middle-distal ileum and cecum with 5 mm submucosal NET (circle). NET = neuroendrocrine tumor.

The multidisciplinary tumor board discussed the case and decided on upfront surgery for three reasons: (a) control of the symptoms, (b) limited efficacy of somatostatin analogs to reduce the mass, and (c) potential risk of mesenteric vascular occlusion if the mass grew. The surgical program included removal of the mass and subrenal interaortocaval and retrocaval lymphadenectomies. We started with a laparoscopic approach but then switched to an open strategy because the mass was larger than expected and difficult to isolate. The mass was removed from the root of the superior mesenteric vessels, occluding the tributary vessels of the middle-distal ileum and cecum; therefore, it was necessary to remove the intestine from the middle ileum to the cecum. On palpation of the bowel, we noted a small submucosal nodule in the middle ileum, that was probably a primitive NET (Fig. 3).

Therefore, we decided against interaortocaval and retrocaval lymphadenectomy for two reasons: (a) risk of further intestinal sacrifice and (b) change of TNM (tumor, node, metastases) from lymph node-positive (N) to distant metastases (M). Indeed, if the primitive tumor originated from the mesentery, the interaortic and retrocaval lymph nodes confer stage N; in our case, the primitive tumor originated from the ileum, and the above-mentioned lymph nodes conferred stage M.

**Figure 4:**
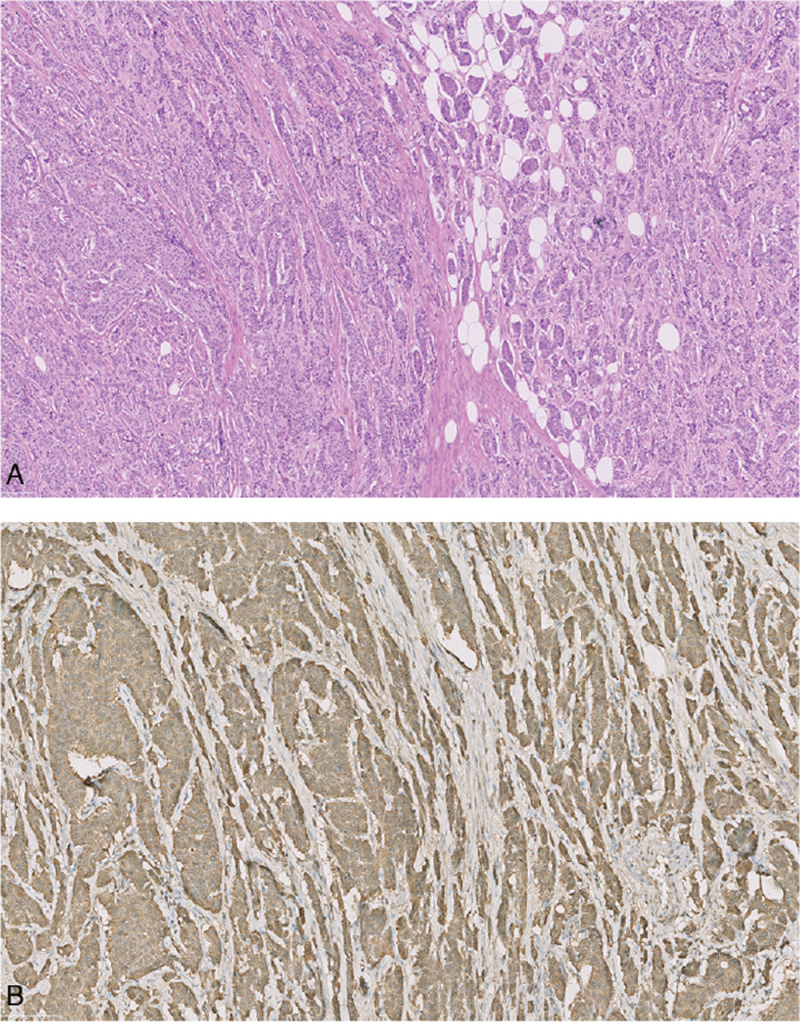
(A) Haematoxylin and eosin staining: neuroendocrine tumor formed by rounded nests of densely packed cells. (B) Immunohistochemical staining for chromogranin A: tumor cells positive for chromogranin A are brown.

The postoperative course was normal, and the patient was discharged on the seventh postoperative day without signs of short bowel syndrome or altered glycemia. Histological examination revealed NET G2 with an ileal primary location (5 mm, Ki 67, 3%), mesenteric lymph node metastases (Ki 67, 9.5%), and pT3N2R0 (TNM VIII edition) (Fig. 4).

After a multidisciplinary tumor board discussion, the oncologists recommended therapy with somatostatin analogs (lanreotide 120 mg every 28 days). After 6 months, the chromogranin A level was 55 μg/L, and Ga PET showed stable lymph nodes. The patient tolerated the somatostatin analogs well, and no longer had altered blood glucose levels. We planned a follow-up program with control of chromogranin A and Ga-PET every 6 months for the first 2 years and then every year.

## 3 Discussion

We report the original case of a patient with preoperative findings of a presumptive PMNET without carcinoid syndrome and stage III disease according to TNM staging. In the literature, PMNETs have been described only in case reports: one with carcinoid syndrome and the other with liver metastases. PMNETs are differentially associated with desmoid tumors, lipomas, lymphomas, schwannomas, smooth muscle tumors, paragangliomas, sarcomas, Castleman disease, metastatic lymphadenopathy, sclerosing mesenteritis, and peritoneal carcinomatosis.^[8,9]^ In general, PMNETs can be detected by the radiological sign (CT scan) of “spoke wheel” given by mesenteric retractions and calcifications.^[10,11]^ However, this pattern was not observed in the present case.

Our multidisciplinary tumor board opted for surgery upfront, and we programmed a complete regional lymphadenectomy along the superior mesenteric vessels according to the North American neuroendocrine tumor society guidelines.^[12,13]^

In primitive intestinal NETs, the risk of lymph node metastasis strongly depends on the primary tumor diameter. If it is ≤2 cm, intestinal segmental resection is indicated because of the low risk of metastasis; if it is ≥ 2 cm, intestinal resection is combined with mesenteric lymphadenectomy because the risk of metastasis is 80%.

Our case is original because there was massive lymph node involvement (mesenteric mass), with no other primitive site of origin identified preoperatively. This is related to the resolving power of Ga-PET, which cannot detect nodules smaller than 5 mm.^[14]^ When operating on NETs, accurate palpation of the entire intestine is essential for two reasons: identifying the primitive site and the possibility of multiple intestinal locations. In addition, frequent presentation to the emergency department and the rarity of the disease increase the risk of inadequate surgical resection. Surgery is generally indicated for locally advanced small intestine NETs and large mesenteric masses that may cause acute or chronic bowel obstruction or ischemia.

There are no data supporting adjuvant chemotherapy for patients with NET G1/G2. In the presence of residual disease or metastatic lymph nodes (interaortocaval and retrocaval), as in our case, therapy with somatostatin analogs was indicated. Chemotherapy is usually indicated for aggressive NETs (NEC G3) in the neoadjuvant and adjuvant settings.^[1]^

## 4 Conclusion

NETs are rare tumors that may occur at different locations in the body. PMNETs are rare, as shown by the few cases described in the literature. Ga PET (DOTATOC or DOTATATE) is a more sensitive and specific imaging modality for detecting this neoplasia, although its resolution is limited. Histological examination remains the cornerstone of diagnosis before any therapeutic decision. Accurate palpation of the intestine is essential during surgery for NET for two reasons: to find the primitive, and because of the risk of multiple intestinal primitives.

## Acknowledgments

The authors acknowledge IRCCS MULTIMEDICA and medical writer Dr. Ferrari Rossella (she gave permission to be named) for their assistance in the preparation of the manuscript.

## Author contributions

Antonio Costanzo, Marco Canziani, Cesare Carlo Ferrari, Valentina Bertocchi, and Fabio Ceriani wrote the abstract, introduction, case, discussion, and conclusions; Saro Cutaia, Eraldo Oreste Bucci, Elisabetta Uslenghi, Andrea Ferretti, and Marco De Luca performed critical edits of the draft and prepared the final version of this manuscript, which was approved by all authors.

**Validation:** Andrea Ferretti
